# Use of Machine Learning Algorithms to Predict the Understandability of Health Education Materials: Development and Evaluation Study

**DOI:** 10.2196/28413

**Published:** 2021-05-06

**Authors:** Meng Ji, Yanmeng Liu, Mengdan Zhao, Ziqing Lyu, Boren Zhang, Xin Luo, Yanlin Li, Yin Zhong

**Affiliations:** 1 School of Languages and Cultures University of Sydney Sydney Australia; 2 School of Foreign Languages Jiangsu University of Science and Technology Zhenjiang China; 3 Department of Chinese and Bilingual Studies The Hong Kong Polytechnic University Hong Kong Hong Kong; 4 The HK PolyU-PKU Research Centre on Chinese Linguistics The Hong Kong Polytechnic University Hong Kong Hong Kong; 5 Department of English The Hong Kong Polytechnic University Hong Kong Hong Kong; 6 Research Centre for Professional Communication in English The Hong Kong Polytechnic University Hong Kong Hong Kong

**Keywords:** machine learning, PEMAT, health education, understandability evaluation, patient-oriented

## Abstract

**Background:**

Improving the understandability of health information can significantly increase the cost-effectiveness and efficiency of health education programs for vulnerable populations. There is a pressing need to develop clinically informed computerized tools to enable rapid, reliable assessment of the linguistic understandability of specialized health and medical education resources. This paper fills a critical gap in current patient-oriented health resource development, which requires reliable and accurate evaluation instruments to increase the efficiency and cost-effectiveness of health education resource evaluation.

**Objective:**

We aimed to translate internationally endorsed clinical guidelines to machine learning algorithms to facilitate the evaluation of the understandability of health resources for international students at Australian universities.

**Methods:**

Based on international patient health resource assessment guidelines, we developed machine learning algorithms to predict the linguistic understandability of health texts for Australian college students (aged 25-30 years) from non-English speaking backgrounds. We compared extreme gradient boosting, random forest, neural networks, and C5.0 decision tree for automated health information understandability evaluation. The 5 machine learning models achieved statistically better results compared to the baseline logistic regression model. We also evaluated the impact of each linguistic feature on the performance of each of the 5 models.

**Results:**

We found that information evidentness, relevance to educational purposes, and logical sequence were consistently more important than numeracy skills and medical knowledge when assessing the linguistic understandability of health education resources for international tertiary students with adequate English skills (International English Language Testing System mean score 6.5) and high health literacy (mean 16.5 in the Short Assessment of Health Literacy-English test). Our results challenge the traditional views that lack of medical knowledge and numerical skills constituted the barriers to the understanding of health educational materials.

**Conclusions:**

Machine learning algorithms were developed to predict health information understandability for international college students aged 25-30 years. Thirteen natural language features and 5 evaluation dimensions were identified and compared in terms of their impact on the performance of the models. Health information understandability varies according to the demographic profiles of the target readers, and for international tertiary students, improving health information evidentness, relevance, and logic is critical.

## Introduction

### Background

The World Health Organization recommends a set of principles for effective health communication, including accessibility, actionability, credibility, relevance, timeliness, and understandability [1]. Health information understandability can be achieved by using familiar language and good writing practice that highlights health information directness, clearness of the desired health outcome, easy-to-follow informational organization, and discourse explicitness, that is, clear explanation of health and medical knowledge using simple, plain, and purposeful language [2-5]. Approaches to health information evaluation can be divided into 2 large categories, that is, expert-led qualitative evaluation based on clinical experiences [6-9] and automated health information analyzers using medical readability formulas or natural language processing tools [10-13]. The strengths and limitations of both approaches are well-known [14-16]. Expert-led health material evaluation draws upon the domain knowledge of medical and health professionals, which are insightful and clinically reliable. This approach, however, is costly and requires much longer evaluation timeframes when compared to automated evaluations. They have important limitations with the evaluation of health materials in large quantities or in situations that require more regular, instant evaluation such as health information updates in health emergencies. Further, this approach is not flexible with user-oriented health information evaluation that requires the evaluation criteria adjust with flexibility to align with the actual reading abilities of the patient education resource users [17,18]. For example, the same piece of health information can be of varying understandability for users with different education levels, health literacy, or existing knowledge of specific health topics. By contrast, the computerized approach of evaluating health information based on natural language features is gaining importance in health informatics [19-22].

Developing health resources of adequate understandability can have important impact on the trust, acceptance, and voluntary adherence to the health advice and recommendations delivered in the health texts [23-26]. Information simplification is an effective strategy to increase the understandability of health materials. However, with specialized health texts, oversimplification can result in critical information loss and reduced believability and persuasiveness of health information for educated readers with higher health information appraisal abilities and health risk assessment autonomies. How to maintain a balance between the understandability and the informativeness of health materials holds the key to optimal health communications. This paper leverages machine learning techniques to develop automated health information evaluation tools of English health materials for a specific group of readers, that is, students in tertiary education from non-English speaking backgrounds with intermediate English reading skills (they achieved an average 6.5 score in the International English Language Testing System test). The stress of living away from home and the adjustment difficulties among international students is known as the “foreign student syndrome” [27,28]. Previous research with students in Australia and internationally showed that international students were less likely to seek for help from health organizations than from residents [29]. English health materials available on the websites of health authorities thus provide important sources of information for international students. Whether and how health information from health authorities developed for native English readers is understandable to international students with intermediate English skills and limited health literacy remains unknown. In this study, new machine learning algorithms were developed to predict the linguistic readability of original English health information for international students. Our study illustrates the training and validation of machine learning algorithms to predict the understandability of health education materials on infectious diseases for this group of English health information users. The strength of machine learning algorithms, that is, adaptiveness and flexibility can significantly improve the cost-effectiveness and efficiency of automated health educational resource evaluation for specific user groups.

The contributions of our study are three-fold: first, we translated clinical health education material evaluation guidelines to machine algorithms to enable the quantitative evaluation of understandability of health materials. This has, for the first time, materialized the automation of health resource understandability assessment with specific reader groups, which represents a significant advance in user-oriented health information evaluation. Second, the results of the machine learning–based evaluation identified important new dimensions in information readability assessment, which are health information purposefulness and the logical structure of health texts. These new findings challenged traditional views that lack of health text readability was caused by morphological complexity and domain-specific terminology. Such views largely simplified the complex issue of the cognitive processing of health information by populations of varying education and health literacy levels and language and cultural backgrounds. Our study shows that for nonnative English readers with tertiary education and high health literacy levels, health information evidentness, logical sequence, and relevance for educational purposes weigh more than health domain knowledge and numeracy demands when assessing the understandability of health texts for readers from similar backgrounds. Lastly, our study identified textual linguistic features having large impact on the performance of machine learning algorithms. For information evidentness, these were words describing mental actions and processes (X2), general/abstract terms (A1), and for relevance to educational purposes, these were words of anatomy, physiology (B1), medicines, and medical treatment (B3). For logical sequence, these were grammatical words (Z5), negative (Z6), and conditional expressions (Z7). Different from statistics, machine learning cannot compute the regression coefficients of these variables within different models, but the large impact of these features on health text readability suggests that linguistic interventions to these features of health texts can significantly improve the performance of machine learning algorithms as automated health text readability evaluation applications.

### Data Sets and Feature Selection

#### Data Collection and Classification

The health educational resources were collected from diverse sources, including governmental health agencies and not-for-profit health organizations in Australia. Health education resource genres are highly diverse, which may be classified into fact sheets, health topics, patient guidelines, clinical guidelines, administrative guidelines, manuals, reports, booklets, brochures, posters, leaflets, checklists, and flipcharts. In this study, we purposely selected health education resources from fact sheets, health topics, and patient guidelines, which are some of the most used health resource varieties. The main sources of credible health information were the Australian Federal and State Health departments and not-for-profit organizations: Arthritis Australia, Australian Food Safety Information Council, Australian Melanoma Research Foundation, Australian Rotary Health, Breast Cancer Network Australia, Cancer Council Australia, Diabetes Australia, National Breast Cancer Foundation, National Heart Foundation of Australia, and National LGBTI Health Alliance. The total corpus contained 1000 full-length health educational texts (running tokens of over 500,000 words). Five international students in tertiary education enrolled in Australian universities classified the collected health texts independently into easy versus hard-to-understand categories (Cohen kappa 0.705). They were aged 25-30 years with advanced English skills (International English Language Testing System test score 6.5 or above). Their mean health literacy level (16.5 [SD 1.69], IQR 13-18) was measured using the Short Assessment of Health Literacy-English [30,31], and their mean level was 87.5% over the threshold 14 of low health literacy.

#### Textual Features as Health Information Understandability Predictors

In order to develop automated health resource evaluation algorithms, we identified a set of key linguistic features as relevant to the understandability of written health resources. Table 1 lists some of the evaluation criteria in the Patient Education Materials Assessment Tool (PEMAT) developed by the Agency for Healthcare Research and Quality, United States Department of Health and Human Services [32]. These include the evaluation of health content, word choice and style, use of numbers, and textual organization. Each evaluation criterion was then mapped onto one or multiple semantic classes of the UCREL English Semantic Analysis System (USAS) developed by the University of Lancaster, United Kingdom [33]. We used USAS to annotate the raw English corpus texts collected. USAS is one of the most used English semantic annotation systems. It has a multi-tier structure with 21 major discourse fields covering (A) general and abstract terms, (B) the body and the individual, (C) arts and crafts, (D) emotion, (E) food and farming, (G) government and public, (H) housing and home, (I) money and commerce, (K) sports and games, (L) live and living things, (M) movement and transport, (N) numbers and measurement, (O) substances, materials, objects, and equipment, (P) education, (Q) language and communication, (S) social actions, states, and processes, (T) time, (W) world and environment, (X) psychological actions, states and processes, (Y) science and technology, and (Z) names and grammars. Within each large semantic category (A-Z), there are subcategories providing fine-grained classification of the word semantics. For example, the A category contains A1 general and abstract terms, A2 affect, A3 being, A4 classification, A5 evaluation, A6 comparison, A7 probability, A8 seem, A9 possession, and so on. These natural language features were then mapped onto the PEMAT evaluation criteria as shown in Table 1.

**Table 1:** Natural language features relevant to Patient Education Materials Assessment Tool guidelines.

Evaluation criteria in the Patient Education Materials Assessment Tool		Language features	Machine learning evaluation
**Content**			
	The material makes its purpose completely evident.	A1, X1, X2, X7	Information evidentness
	The material does not include information that distracts from its purpose.	B1, B3	Relevance to education purpose
**Word choice and style**			
	Medical terms are used only to familiarize audience with the terms.	B2	Domain knowledge
**Use of numbers**			
	The material does not expect the user to perform calculations.	N1, N2, N3	Numeracy demand
**Organization**			
	The material presents information in a logical sequence.	Z5, Z6, Z7	Logical sequence

To quantify the PEMAT guideline item “the material makes its purpose completely evident,” 4 USAS classes were used as quantitative measures, that is, A1: general and abstract terms; X1: psychological actions, states, and processes; X2: mental actions and processes (such as think, analyze, study, look over, go over); and X7: wanting, planning, choosing (such as aim, objective, goal, target, intention, purpose, plan, idea, point). To quantify the PEMAT guideline item “the material does not include information or content that distracts from its purpose,” 2 USAS classes were used as quantitative measures, that is, B1: anatomy and physiology and B3: medicines and medical treatment. Typical examples of content distraction include excessive detail about the equipment used for a procedure that distracts from the material’s purpose or excessive detail about other procedures or treatments that are not related to the material’s purpose. To quantify the PEMAT guideline item “medical terms are used only to familiarize audience with the terms,” the USAS class B2: health and disease terms were used as the main quantitative measure. To quantify the PEMAT guideline item “the material does not expect the user to perform calculations,” 3 USAS classes were selected from the USAS semantic tag set as relevant quantitative measures. To quantify the PEMAT guideline item “the material presents information in a logical sequence,” 3 USAS classes were identified as relevant to the logical structure of health materials, that is, Z5: grammatical bin, Z6: negative, and Z7: if (conditional). In total, 13 semantic annotation classes were selected from the extensive tag set of USAS. Information evidentness of written health texts is measured by A1, X1, X2, X7; information relevance to educational purposes by B1 and B3; health domain knowledge by B2; health numeracy demand by N1, N2, and N3; and lastly, text logical sequence by grammatical and functional features Z5, Z6, and Z7.

### Analysis of the Differences Between Easy and Difficult Texts

Table 2 shows the statistical results of the differences between easy and difficult health educational texts for international college students. All the predictor variables were continuous variables, and the *P* values were derived using Mann-Whitney *U* test. The result shows that statistically significant differences (*P*<.05) exist in most of the semantic features. Easy and difficult health texts, however, did not differ significantly in the semantic classes of B1 (anatomy, physiology), N1 (numbers), N2 (mathematics), and N3 (measurement). The mean values of the 7 semantic classes of easy health texts were significantly higher than those of difficult health texts. In terms of health information purposefulness, 4 semantic features contributed to the linguistic understandability of health resources, that is, A1 (14.09 easy vs 10.10 difficult), X1 (0.42 easy vs 0.18 difficult), X2 (10.41 easy vs 6.57 difficult), and X7 (3.24 easy vs 1.79 difficult). This suggests that the increased use of words describing the psychological and mental actions, states, and processes can help the target readers to understand the textual information. A1 is defined as general and abstract words.

**Table 2:** Differences between easy and difficult medical texts derived by the Mann-Whitney *U* test.

Variables	Easy texts, mean (SD) score	Difficult texts, mean (SD) score	Mann-Whitney *U*	*P* value
A1	14.09 (14.52)	10.10 (13.13)	97905.00	<.001
X1	0.42 (3.89)	0.18 (1.49)	120325.50	.02
X2	10.41 (11.26)	6.57 (9.14)	89487.50	<.001
X7	3.24 (5.41)	1.79 (3.12)	103350.50	<.001
B1	17.10 (31.14)	15.69 (21.78)	117882.50	.12
B2	15.04 (21.53)	24.68 (34.04)	99536.50	<.001
B3	9.25 (14.30)	12.80 (18.02)	103338.00	<.001
N1	5.74 (8.54)	5.42 (6.51)	123009.00	.66
N2	0.21 (0.70)	0.21 (0.70)	123284.50	.52
N3	5.73 (9.38)	4.77 (5.60)	120978.50	.38
Z5	133.63 (118.93)	122.77 (119.05)	108744.00	<.001
Z6	4.13 (5.28)	3.01 (5.01)	100719.00	<.001
Z7	4.22 (4.62)	2.10 (4.03)	81063.50	<.001

Table 3 shows some of the words annotated as A1 in a typical health text classified as difficult. These general and abstract words were not typical medical and health terms. They were classified and tagged in the corpus study as general English terms. However, the statistically significant *P* value attributed to this word category as shown in Table 2 indicated that they can be used as a discriminating feature to separate easy versus difficult health educational materials for international students in tertiary education. Regarding health domain knowledge, the result shows that the mean of B2 (health and disease) of easy health texts (15.04) was significantly lower than that of difficult texts (24.68). In terms of numeracy demand, the 2 sets of health texts did not different significantly, suggesting that for international students in tertiary education, the use of numbers and quantitative measures in health educational texts did not represent an important barrier. Lastly, the logical sequence of English health texts can be improved using functional words (Z5, Z6, Z7), as the mean scores of these 3 linguistic features in easy health educational resources proved to be significantly higher than those of difficult texts: Z5 (133.63 easy, 122.77 difficult), Z6 (4.13 easy, 3.01 difficult) and Z7 (4.22 easy, 2.10 difficult).

**Table 3:** A1 in difficult texts.

A1	Keyword concordances
limited to	infections in humans are *limited* to one case of Taï Forest Ebola virus
strains	There are five *strains* that have been identified: Zaire, Sudan, Bundibugyo, Taï Forest, and Reston.
containment	Previous outbreaks had been limited to remote areas allowing initial *containment* efforts to be more effective.
combined	This outbreak was unprecedented in scale, being larger than all other outbreaks *combined.*
spread	The virus *spread* across multiple international boundaries.
boundaries	The virus spread across multiple international *boundaries.*
isolated	Seven other countries had minor outbreaks with nonsustained transmission or *isolated* cases.
events	This article aims to summarize the *events* by country in chronological order.

## Methods

### Machine Learning Algorithms

The 5 machine learning methods used in this study were extreme gradient boosting (XGBoost) tree, random forest, deep neural networks, and C5.0 decision tree. Logistic regression was used as the baseline model for the evaluation of the performance of the 5 machine learning models. Both XGBoost and random forest are ensemble learning techniques that can be used for both classification and regression issues. Ensemble learning can boost the predictive performance of a single learning algorithm, which is merely better than random guesses. Random forest uses bagging or bootstrap to combine base learners to significantly improve the prediction of the model. XGBoost uses gradient boosting to combine decision trees as base learners. The C5.0 decision tree is a typical tree-based machine learning algorithm. XGBoost, random forest, and C5.0 can be used to learn any patterns underlying the training data without implicit assumptions of the data profiles, such as distribution normality, nonlinearity, multi-linearity, or higher order interactions between the variables. The type of neural networks used in this study is multilayer perceptron, which is a class of feedforward artificial neural network. This technique has been used to provide a nonlinear mapping between the input vector and the output vector. Between the input and output layers, there could be an arbitrary number of hidden layers, which perform complex computations. The strength of multilayer perceptron is to map nonlinear relations between input features and outcomes. The major uses of multilayer perceptron are pattern classification, recognition, prediction, and approximation. The research work of this paper can be seen as a text classification task. Random forest is suitable for analyzing data of high dimensions, as the algorithm builds separate trees and uses bootstrapping to combine these tree-based single learners trained on random subsets of input features. Like random forest, gradient boosting tree is a type of supervised learning algorithm known for its high prediction accuracy.

### Hyperparameters of Machine Learning Algorithms

In this study, hyperparameter tuning of XGBoost involved the following steps. The maximum tree depth for base learners (max_depth) controls the depth of the tree. The larger the depth, the more complex is the model, and the higher are the chances of model overfitting. There is no standard value for max_depth. Larger data sets require deep trees to learn the rules from a complex data set. The value ranges between 0 and infinite. In the cross-validation process, we set max_depth to the default value 8. The number of estimators or boosted trees was set to the default value 20. The minimum sum of instance weight needed in a child node (min_child_weight) is another effective overfitting prevention method. It is calculated by second-order partial derivatives and ranges between 0 and infinite. The larger the value, the more conservative the algorithm is. This was set to the default value of 1 in this study. The maximum delta step (max_delta_step) specifies the maximum step size that a leaf node can take. It ranges between 0 and infinite. Increasing the positive value will make the update step more conservative. The learning objective was set to binary logistic regression, as the target variable has 2 outcome categories, that is, easy versus difficult health education texts. Subsample refers to the subsample ratio of the training instance. For example, setting a subsample to 0.5 means that the algorithm randomly collects half of the entire data set to build the tree model. The value of the subsample was set to the default value 1. Eta refers to the machine learning rate at which the algorithm learns the latent patterns and structures in the training data set. Smaller eta leads to slower computation and thus prevents overfitting. Smaller etas can be compensated by increasing the number of boosted trees or estimators; 0.6 was set as the value in this study. The hyperparameter colsample_bytree controls the number of features or variables supplied to a tree model. It was set to 1. Lastly, alpha and lambda values, which control L1 and L2 regularization, respectively, were set to 1 and 0 to prevent overfitting. Random forest is another powerful ensemble learning technique that outperforms single learning algorithms in machine learning model development. In random forest, decision trees are used as the base learner and bootstrapping aggregation combines these decision trees together to achieve high prediction accuracy. The minimum number of samples and training data required to be at a leaf node (min_samples_leaf) was set to 1. The maximum depth was set to 10. The number of features to use for splitting was set to auto. In the model construction process, the ensemble learning methods selected to increase the prediction accuracy included bootstrapping, bagging, and extremely randomized trees. In the process of hyperparameter optimization, on each iteration, the algorithm will choose a different combination of the features. The maximum number of iterations was set to 1000, and the maximum evaluations were set to 300. The neural networks model used in this study is multilayer perceptron. Only one hidden layer was configured, which contained 13 nodes as the input features (Table 1). The overfitting prevention rate was set to 30%.

## Results

### Predictive Performance Evaluation

**Figure 1:**
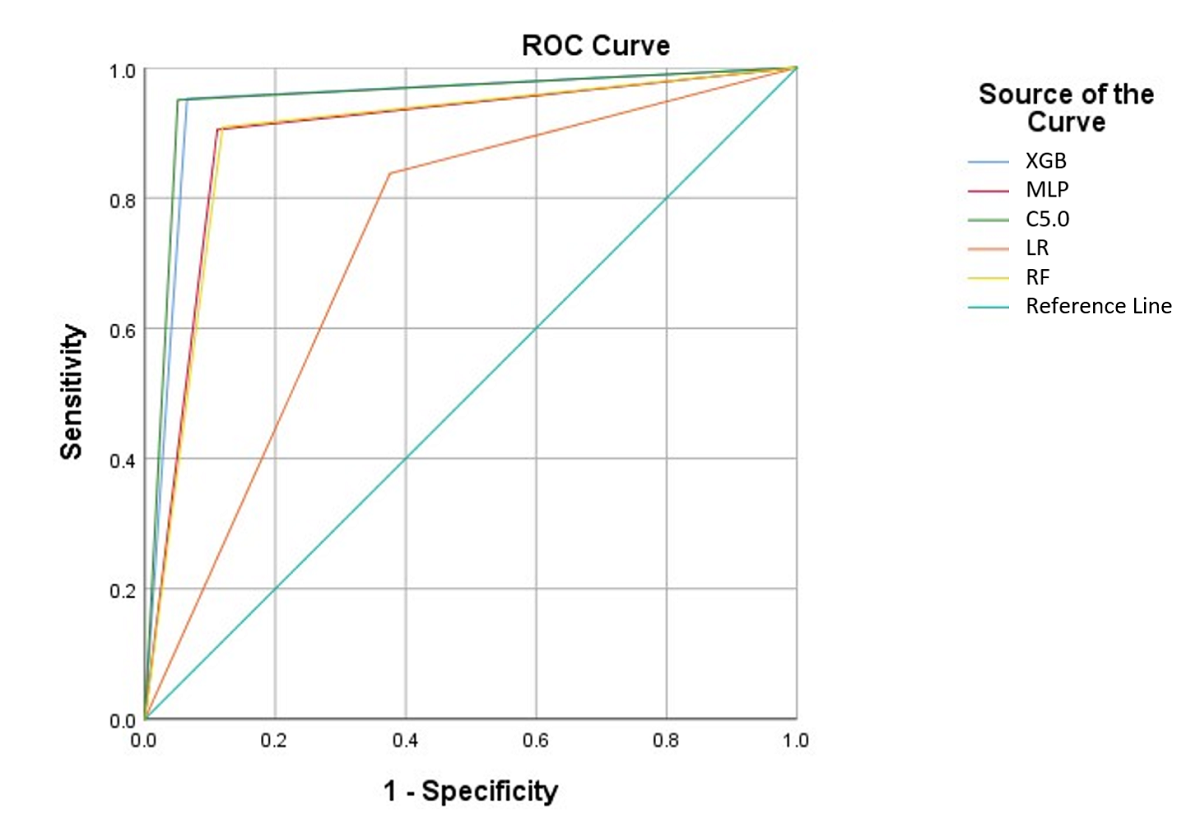
Mean receiver operating characteristic curve for the 5 machine learning algorithms. C5: C5 decision tree; LR: logistic regression; MLP: multilayer perceptron; ROC: receiver operating characteristic; RF: random forest; XGB: extreme gradient boosting.

The predictive performance of the 5 machine learning algorithms is shown in Figure 1 and Table 4, and the results of the pairwise corrected resampled two-tailed *t* test are shown in Table 5. The mean scores and their standard deviations of area under the receiver operating characteristic curve (AUC), sensitivity, specificity, and accuracy were obtained through five-fold cross-validation. The cross-validation divided the entire data set into 5 folds of equal size. In each iteration, 5 folds were used as the training data and the remaining fold as the testing data. As a result, on completion of the five-fold cross-validation, each fold was used as the testing data exactly once. We used the pairwise corrected resampled *t* test to counteract the issue of multiple comparisons. The significance level was adjusted to .005 using Bonferroni correction.

**Table 4:** Performance of the 5 machine learning models on predicting language understandability of the health texts for international students in tertiary education.

Algorithm	Area under the receiver operating characteristic curve, mean (SD)	Sensitivity, mean (SD)	Specificity, mean (SD)	Accuracy, mean (SD)
Extreme gradient boosting	0.979 (0.006)	0.947 (0.011)	0.944 (0.011)	0.945 (0.01)
Random forest	0.967 (0.033)	0.924 (0.034)	0.885 (0.094)	0.904 (0.064)
Multilayer perceptron	0.946 (0.006)	0.897 (0.006)	0.893 (0.014)	0.895 (0.008)
C5.0 decision tree	0.981 (0.005)	0.95 (0.009)	0.941 (0.023)	0.945 (0.014)
Logistic regression	0.804 (0.002)	0.837 (0.009)	0.627 (0.016)	0.732 (0.004)

The 5 machine learning models (ie, XGBoost, random forest, multilayer perceptron, and C5.0 decision tree) achieved significantly higher AUCs than the linear logistic regression algorithm: XGBoost (*P*<.001), random forest (*P*<.001), C5.0 (*P*<.001), multilayer perceptron (*P*<.001) (Table 4). To be more specific, C5.0 decision tree had a mean score of 0.981 in terms of AUC, followed by XGBoost (0.979), random forest (0.967), neural networks (0.946), and logistic regression (0.804). XGBoost and C5.0 had significantly higher AUC (Table 5) than multilayer perceptron (XGBoost vs MLP, *P*<.001; MLP vs C5.0, *P*=.001), whereas no significant differences were found between the mean AUCs of XGBoost, random forest, and C5.0 decision tree (XGBoost vs RF, *P*=.44; XGBoost vs C5.0, *P*=.66; RF vs C5.0, *P*=.34). Similarly, all 5 machine learning algorithms had significantly higher mean sensitivity scores than the baseline logistic regression (*P*=.005). C5.0 had the highest mean sensitivity score (0.95) followed by XGBoost (0.947), random forest (0.924), neural networks (0.897), and logistic regression (0.837). XGBoost and C5.0 achieved significantly higher sensitivity scores than multilayer perceptron (XGBoost vs MLP, *P*<.001; MLP vs C5.0, *P*<.001), whereas no significant differences were found between the mean sensitivity scores of XGBoost, C5.0 decision tree, and random forest (XGBoost vs C5.0, *P*=.40; RF vs C5.0, *P*=.15; XGBoost vs RF, *P*=.21). With regards to specificity, that is, the ability of the models to accurately identify health texts classified as easy health education resources, the 5 machine learning models outperformed logistic regression (XGBoost vs LR, *P*<.001; RF vs LR, *P*=.003; MLP vs LR, *P*<.001; C5.0 vs LR, *P*<.001). Again, the mean specificity score of multilayer perceptron was significantly lower than that of XGBoost tree (XGBoost vs MLP, *P*=.001), but not significantly lower than C5.0 (MLP vs C5.0, *P*=.01) and random forest (RF vs MLP, *P*=.86) at the adjusted .005 significance level using Bonferroni correction. Lastly, in terms of overall accuracy, XGBoost and C5.0 achieved the highest mean scores of 0.945, followed by random forest (0.904) and neural networks (0.895). These scores were significantly higher than the mean overall accuracy of logistic regression (0.732) (XGBoost vs LR, *P*<.001; RF vs LR, *P*=.003; MLP vs LR, *P*<.001; C5.0 vs LR, *P*<.001). Again, the differences in the model accuracy were insignificant among XGBoost, C5.0, and random forest (XGBoost vs C5.0, *P*>.99; XGBoost vs RF, *P*=.21; RF vs C5.0, *P*=.17), but significant between the 2 best performing models (XGBoost vs MLP, *P*<.001; MLP vs C5.0, *P*=.002).

**Table 5:** Results of the pairwise comparison of the model predictive performance by two-tailed *t* test.

Pair number	Comparison	AUC^a^ difference			Sensitivity difference			Specificity difference			Accuracy difference	
		Mean (SD)	*P* value	Mean (SD)		*P* value	Mean (SD)		*P* value	Mean (SD)		*P* value
Pair 1	XGB^b^ vs RF^c^	0.013 (0.034)	.44	0.023 (0.034)		.21	0.059 (0.089)		.21	0.041 (0.062)		.21
Pair 2	XGB vs MLP^d^	0.034 (0.007)	<.001^e^	0.049 (0.009)		<.001^e^	0.051 (0.013)		.001^e^	0.050 (0.010)		<.001^e^
Pair 3	XGB vs C5.0	–0.001 (0.006)	.66	–0.003 (0.008)		.40	0.003 (0.022)		.76	0.000 (0.015)		>.99
Pair 4	XGB vs LR^f^	0.175 (0.004)	<.001^e^	0.109 (0.006)		<.001^e^	0.317 (0.021)		<.001^e^	0.213 (0.012)		<.001^e^
Pair 5	RF vs MLP	0.021 (0.036)	.27	0.026 (0.037)		.19	–0.008 (0.095)		.86	0.009 (0.066)		.77
Pair 6	RF vs C5.0	–0.014 (0.029)	.34	–0.026 (0.032)		.15	–0.056 (0.076)		.18	–0.041 (0.054)		.17
Pair 7	RF vs LR	0.163 (0.033)	<.001^e^	0.086 (0.034)		.005^e^	0.258 (0.090)		.003^e^	0.172 (0.062)		.003^e^
Pair 8	MLP vs C5.0	–0.035 (0.008)	.001^e^	–0.052 (0.011)		<.001^e^	–0.048 (0.023)		.01	–0.050 (0.016)		.002^e^
Pair 9	MLP vs LR	0.142 (0.005)	<.001^e^	0.060 (0.007)		<.001^e^	0.266 (0.015)		<.001^e^	0.163 (0.008)		<.001^e^
Pair 10	C5.0 vs LR	0.177 (0.004)	<.001^e^	0.112 (0.010)		<.001^e^	0.314 (0.020)		<.001^e^	0.213 (0.014)		<.001^e^

^a^AUC: area under the receiver operating characteristic curve.

^b^XGB: extreme gradient boosting.

^c^RF: random forest.

^d^MLP: multilayer perceptron.

^e^Significant at the adjusted .005 significance level using Bonferroni correction.

^f^LR: logistic regression.

### Variable Ranking

**Figure 2:**
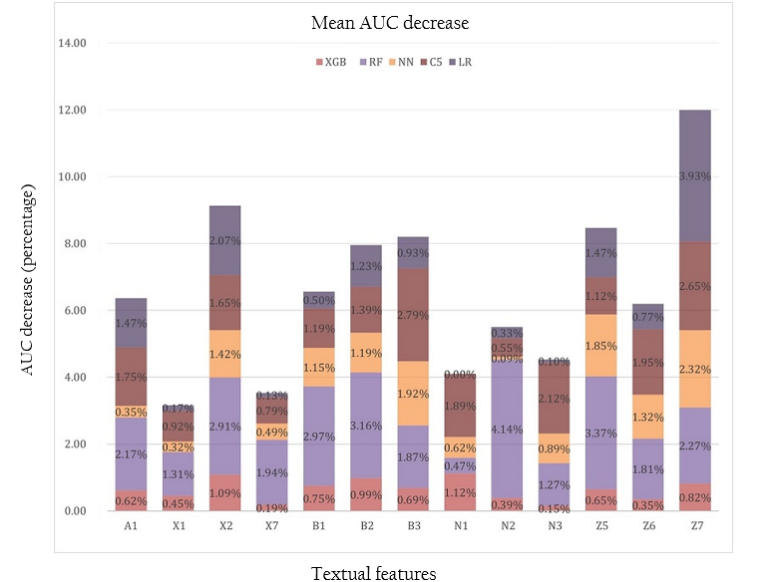
The impact of different linguistic features on the machine learning algorithms. AUC: area under the receiver operating characteristic curve; C5: C5 decision tree; LR: logistic regression; NN: neural networks; RF: random forest; XGB: extreme gradient boosting. A1: general and abstract terms; X1: psychological actions, states, and processes; X2: mental actions and processes; X7: wanting, planning, and choosing; B1: anatomy and physiology; B2: health and disease; B3: medicines and medical treatment; N1: numbers; N2: mathematics; N3: measurement; Z5: grammatical bin; Z6: negative; Z7: if.

To have a deeper understanding of the 5 machine learning algorithms, including the baseline logistic regression, we ranked the impact of the 13 predictor variables on the mean AUCs of the 5 algorithms. This was achieved through the successive permutation of the values of the input linguistic features. To ensure the stability and reliability of the experimental models, five-fold cross-validation was repeated with each permutation exercise. As a result, we obtained the mean decease (in percentage) in the AUCs of the 5 machine learning algorithms as shown in Table 6 and Figure 2.

**Table 6:** Mean decrease in the area under the receiver operating characteristic curve of the 5 machine learning algorithms.

Feature	Predictor variable	Extreme gradient boosting (%)	Random forest (%)	Deep neural networks (%)	C5.0 decision tree (%)	Logistic regression (%)
General and abstract terms	A1	0.62	2.17	0.35	1.75	1.47
Psychological actions, states, processes	X1	0.45	1.31	0.32	0.92	0.17
Mental actions and processes	X2	1.09	2.91	1.42	1.65	2.07
Wanting, planning, and choosing	X7	0.19	1.94	0.49	0.79	0.13
Anatomy and physiology	B1	0.75	2.97	1.15	1.19	0.50
Health and disease	B2	0.99	3.16	1.19	1.39	1.23
Medicines and medical treatment	B3	0.69	1.87	1.92	2.79	0.93
Numbers	N1	1.12	0.47	0.62	1.89	0.00
Mathematics	N2	0.39	4.14	0.09	0.55	0.33
Measurement	N3	0.15	1.27	0.89	2.12	0.10
Grammatical bin	Z5	0.65	3.37	1.85	1.12	1.47
Negative	Z6	0.35	1.81	1.32	1.95	0.77
If	Z7	0.82	2.27	2.32	2.65	3.93

The results showed that for the best performing algorithm, that is the XGBoost tree, linguistic features that had relatively larger impact on the mean model AUC were N1 (numbers, 1.12%), X2 (mental actions and processes, 1.09%), B2 (health and disease, 0.99%), and Z7 (if, conditional, 0.82%). Textual features that had relatively large impact on C5.0 decision tree were B3 (medicines and medical treatment, 2.79%), Z7 (if, conditional, 2.65%), N3 (measurements, 2.12%), Z6 (negative, 1.95%), N1 (numbers, 1.89%), A1 (general and abstract terms, 1.75%), X2 (mental actions and processes, 1.65%), B2 (health and disease, 1.39%), B1 (anatomy and physiology, 1.19%), and Z5 (grammatical bin, 1.12%). For random forest, most linguistic variables had impact on the decrease of the mean AUC larger than 1% and the only exception was N1 (numbers), which reduced the AUC by 0.47%. For the baseline logistic regression, 5 linguistic features reduced the model AUC by more than 1.0%: Z7 (if, conditional, 3.93%), X2 (mental actions/processes, 2.07%), A1 (general and abstract terms, 1.47%), Z5 (grammatical bin, 1.47%), and B2 (health and disease, 1.23%).

### AUC Impact of Individual Textual Features

It is worth noting that the AUC impact of these linguistic features on each of the 5 machine learning algorithms did not correlate with their significance to discriminate between easy and difficult health texts. For example, Table 2 shows that there were no statistically significant differences between easy and difficult health texts in their means of B1 (anatomy and physiology, *P*=.12), N1 (numbers, *P*=.66), N2 (mathematics, *P*=.52), and N3 (measurement, *P*=.38). As a result, these features had limited impact on the mean AUC of logistic regression. By contrast, B1 had large impact on the mean AUC of random forest (2.97% AUC decrease), C5.0 (1.19% AUC decrease), and neural networks (1.15% AUC decrease); N1 had large impact on the AUC of XGBoost (1.12% AUC decrease) and C5.0 (1.89% AUC decrease); N2 had large impact on random forest (4.14% AUC decrease) and N3 had large impact on random forest (1.27% AUC decrease) and C5.0 (2.12% AUC decrease). It became clear that XGBoost was the most parsimonious model that achieved the highest mean AUC with less textual features as large predictor variables. The 4 linguistic features with large impact on the AUC of XGBoost, N1, X2, B2, Z7 suggest that 5 evaluation dimensions were critical to the quantitative analysis of the understandability of health education resources.

### Impact of the 5 Evaluation Dimensions on the Algorithm Performance (AUCs)

As shown in Table 7, for XGBoost, the evaluation dimension that had the largest impact on the AUC of the algorithm was information evidentness (2.35%), followed by information in logical sequence (1.82%), numeracy skills (1.66%), and the relevance of health information for educational purposes (1.44%). Medical domain knowledge was ranked as the dimension with the least AUC impact (0.99%). Similar patterns were found with random forest. Health information evidentness (8.33%) was ranked as the most impactful dimension, followed by textual logical sequence (7.45%), numeracy skills (5.88%), and the relevance of health information for educational purposes (4.84%). Again, medical knowledge (3.16%) had the smallest impact on the AUC of random forest. C5.0 decision tree differs from XGBoost tree and random forest in that logical sequence (5.72%) replaced information evidentness (5.11%) as the dimension with the largest impact on the C5.0 tree model. Neural networks identified logical sequence (5.49%), relevance to health educational purposes (3.07%), and information evidentness (3.07%) as the 3 evaluation dimensions with the largest impact on the model performance, followed by numeracy skills (1.6%) and domain knowledge (1.19%). Similar to the first 4 machine learning algorithms, logistic regression also identified logical sequence (6.17%) as the most impactful dimension on the model performance, followed by information evidentness (3.84%), educational relevance (1.43%), domain knowledge (1.23%), and numeracy skills (0.43%). It is useful to note that for all models, logical sequence, information evidentness, and educational purpose relevance were identified as the most important dimensions with the largest impact on the model prediction accuracy, whereas medical domain knowledge was ranked as the dimension with the least impact on the algorithm performance.

**Table 7:** Impact of the different dimensions on the area under the curves of the algorithms.

Evaluation dimensions	Understandability	Predictor variable	Extreme gradient boosting (%)	Random forest (%)	Deep neural networks (%)	C5.0 decision tree (%)	Logistic regression (%)
Dimension 1	Information evidentness	A1, X1, X2, X7	2.35	8.33	2.58	5.11	3.84
Dimension 2	Relevance to education purpose	B1, B3	1.44	4.84	3.07	3.98	1.43
Dimension 3	Domain knowledge	B2	0.99	3.16	1.19	1.39	1.23
Dimension 4	Numeracy demand	N1, N2, N3	1.66	5.88	1.60	4.56	0.43
Dimension 5	Logical sequence	Z5, Z6, Z7	1.82	7.45	5.49	5.72	6.17

## Discussion

### Principal Findings

The study of the readability of health educational resources has, for long, relied on medical readability calculators among which the Flesch Reading Ease Score [34], Gunning Fog [35], Flesch-Kincaid Grade Level Readability [34], Coleman-Liau Index [36], Simple Measure of Gobbledygook Index [37], Automated Readability Index [38], and Lensear Write Formula [39] are some of the most influential and widely used ones. However, this medical formula–based approach to linguistic readability evaluation, despite being convenient and fast, has known limitations, including interformula inconsistency and reported lack of flexibility and adaptability with populations with diverse language, cultural backgrounds, as well as cognitive abilities. Furthermore, these evaluation tools were originally designed for readers from native English-speaking backgrounds, assuming the health educators who developed the health resources and the target readers have similar knowledge and understanding of the general English vocabulary, logical organization of health materials, and communication of the intentions and purposes of health educational materials. These assumptions, which underlined the design of existing medical readability formula, were increasingly challenged by applications of these tools with diverse populations and communities with limited exposure to the health care systems of English-speaking countries [40-42]. The limitation of the existing medical readability tools also reflects in their exclusive focus on the morphological, syntactic complexity, using low-frequency polysyllabic words, medical terminology, and sentence lengths as the main textual complexity measures.

The more recent patient-oriented health resource evaluation guidelines such as PEMAT has greatly enriched the dimensions of readability evaluation, expanding the evaluation criteria from medical domain knowledge (using familiar, everyday language) to encompass dimensions such as health information relevance, purposefulness to the target readers (information classified as distractor or key information), numeracy demand, and the logical sequence of health texts. Despite the wide adoption of these more comprehensive and user-adaptive evaluation guidelines, no quantitative tools have been developed to implement the multidimensional evaluation in a cost-effective, instant manner. This represents a critical research gap in current health material evaluation, as there are growing demands from both clinical and research settings for automated evaluation tools of the understandability of written health materials. Advances in computational methods such as machine learning algorithms can help address the increasing gap between the practical needs for more cost-effective, integrated quantitative tools that are able to deal with health texts in large quantities and the known limitations of medical readability formulas and expert-led evaluation guidelines, which are slow and time-consuming to implement and update.

Our study developed the first quantitative tool for the evaluation of written health education materials based on the PEMAT guidelines. We developed and compared 5 machine learning algorithms by using logistic regression as the baseline model. The results showed that all 5 models (XGBoost, C5.0, random forest, multilayer perceptron) outperformed logistic regression in terms of AUC, sensitivity, specificity, and overall accuracy. We found that in the evaluation of health information understandability, information evidentness, educational relevance, and logical sequence were ranked consistently more important than numeracy skills and medical domain knowledge. This ranking of the importance of these evaluation dimensions may be explained by the demographical profiles of the target readership: international students in tertiary education with adequate English skills (International English Language Testing System mean score 6.5) and high health literacy (mean score 16.5 in the Short Assessment of Health Literacy-English test). These results challenged the traditional view that lack of medical knowledge and numeracy skills caused the lack of health information understandability. Improving the writing style and health information organization can significantly improve the understandability of health information for non-English speakers, especially for those of higher educational attainment and health literacy levels and with distinct language and cultural backgrounds.

### Limitations and Future Research

The textual linguistic features used in the model development were limited. In future research, we will increase the features to be studied in the evaluation of health material understandability, by adding, for example, syntactic and morphological features of texts. The underlying evaluation framework we used was PEMAT. There are, however, other studies that explored health information accessibility from cognitive and psychological experiments. These studies may help expand the current scope of PEMAT, which is intended for the evaluation of written health resources for readers with average cognitive skills, rather than those with cognitive impairments caused by physical or mental health issues. The new quantitative tools have the potential to be further adapted for different readerships as well as written health materials in languages other than English.

### Conclusions

An important contribution of this paper lies in its efforts to bridge the gap between the 2 distinct approaches to health information evaluation. This was achieved via the translation of clinically developed patient health education materials assessment guidelines to quantitative evaluation models, that is, machine learning algorithms by using a limited number of semantic features to accurately predict the readability (binary outcome) of health educational resources for international students in tertiary education with adequate English proficiency and health literacy but distinct language and cultural backgrounds.

## Abbreviations

AUC: area under the receiver operating characteristic curve
PEMAT: Patient Education Materials Assessment Tool
USAS: UCREL English Semantic Analysis System
XGBoost: extreme gradient boosting
